# “Neural overlap of L1 and L2 semantic representations across visual and auditory modalities: a decoding approach”

**DOI:** 10.1016/j.neuropsychologia.2018.03.037

**Published:** 2018-05

**Authors:** Eowyn Van de Putte, Wouter De Baene, Cathy J. Price, Wouter Duyck

**Affiliations:** a Department of Experimental Psychology, Ghent University, Ghent, Belgium; b Department of Cognitive Neuropsychology, Tilburg University, Tilburg, the Netherlands; c Wellcome Centre for Human Neuroimaging, Institute of Neurology, UCL, London, UK

**Keywords:** mvpa, production, comprehension, word reading, word listening, neural overlap, semantic, fMRI, bilingualism

## Abstract

This study investigated whether brain activity in Dutch-French bilinguals during semantic access to concepts from one language could be used to predict neural activation during access to the same concepts from another language, in different language modalities/tasks. This was tested using multi-voxel pattern analysis (MVPA), within and across language comprehension (word listening and word reading) and production (picture naming). It was possible to identify the picture or word named, read or heard in one language (e.g. *maan*, meaning *moon*) based on the brain activity in a distributed bilateral brain network while, respectively, naming, reading or listening to the picture or word in the other language (e.g. *lune*). The brain regions identified differed across tasks. During picture naming, brain activation in the occipital and temporal regions allowed concepts to be predicted across languages. During word listening and word reading, across-language predictions were observed in the rolandic operculum and several motor-related areas (pre- and postcentral, the cerebellum). In addition, across-language predictions during reading were identified in regions typically associated with semantic processing (left inferior frontal, middle temporal cortex, right cerebellum and precuneus) and visual processing (inferior and middle occipital regions and calcarine sulcus). Furthermore, across modalities and languages, the left lingual gyrus showed semantic overlap across production and word reading. These findings support the idea of at least partially language- and modality-independent semantic neural representations.

## Highlights

• Evidence for at least partially language- and modality-independent semantic neural representations.
• With a decoding approach, we tested whether brain activity during the semantic access of individual nouns in one language and modality (e.g. production) allowed predicting the semantic access of the same concepts in the other language and modalities (e.g. word listening, word reading).
• Across modalities and languages, the left lingual gyrus showed semantic overlap across production and word reading.

The representation of semantics in the brain is a fundamental prerequisite to understand human nature and the creation of meaning. A part of this debate relates to how the semantic system is differently organized and recruited across different language modalities, such as reading, speaking or listening. Several studies have highlighted the existence of amodal conceptual representations (Bright et al., 2004, Buckner et al., 2000, Kircher et al., 2009, Pobric et al., 2010) assuming a semantic system that is shared across modalities. The reviews of Barsalou et al. (2003) and Kiefer and Pulvermüller (2012), however, indicate that concepts may also be flexible, distributed in the brain, and dependent on language modality and the specific sensory and motor characteristics involved. An attempt to reconcile these views was offered by Bonner et al. (2013), who assumed a distributed semantic network that includes an amodal, integrative representation and sensory and motor feature representations in modality-specific association areas. However, most fMRI studies of the representation of semantics have investigated language comprehension and production separately, using different experimental designs and tasks that also rely on additional orthographical or phonological processing to a varying degree. As a consequence of this heterogeneity in tasks, a large variety of brain regions have been reported during semantic language processing, often without very explicit delineation of the processes involved in the investigated tasks. Given that the different tasks and modalities, and the underlying cognitive processes, might recruit distinct neural structures, this paradigmatic diversity may confound conclusions about the neural representation of semantics. Binder et al. (2009) therefore reviewed 120 classical functional neuroimaging studies, rigorously selected on well-defined task contrasts focusing on the neural representation of the semantic system in word reading and word listening in the first language (L1), without additional phonological or orthographic confounds. They concluded that semantic processing occurred in a distributed network including prefrontal, parietal and temporal areas. They highlighted the role of these regions in the representation of amodal conceptual knowledge where information from different modalities is integrated.

However, brain areas that are commonly activated in different language tasks (e.g. picture naming, written word reading, listening to spoken words) do not necessarily represent amodal conceptual information. In the classical univariate fMRI approach, activation in a common brain area in different modalities does not necessarily imply that the semantic representations overlap across the different modalities. More specifically, activation in common brain areas may reflect either different semantic representations for the different modalities or amodal semantic representations. However, within this classical fMRI approach a distinction between these two possibilities can not be made.

Here, multi-voxel pattern analysis (MVPA) may be very useful for a more fine-grained analysis of the overlap of semantic representations across modalities (Haynes et al., 2007, Norman et al., 2006). In MVPA, it is only possible to predict or classify a given concept across different modalities if semantic representations overlap across modalities. Semantic overlap across different modalities is rarely investigated through MVPA and to our knowledge it has only been applied to monolingual (L1) language processing (Fairhall and Caramazza, 2013, Shinkareva et al., 2011, Simanova et al., 2014). Shinkareva et al. (2011) were the first to study semantic processing in L1 using MVPA. In this study, participants saw words and pictures from two semantic categories and they were instructed to consistently think about the properties of the concepts. They showed that the category of the picture the participant was viewing could be predicted based on the neural activation patterns associated with the corresponding written word form and vice versa. More accurate decoding was possible independent of the stimulus format (pictures/words) in the fusiform gyrus, precuneus, paracentral lobule, superior parietal lobule, inferior and superior extrastriate cortex, intraparietal sulcus, supplementary motor area, posterior cingulate, postcentral and precentral gyri, and posterior superior and inferior temporal gyri. In addition to the shared brain regions across modalities, Shinkareva et al. (2011) also provided evidence for modality-specific neural activation in the pars opercularis and pars triangularis. In a later study by Fairhall and Caramazza (2013), participants saw words and pictures from five semantic categories and they needed to judge how typical each item was for the representation of its semantic category. The authors argued that the precuneus and the posterior middle/inferior temporal gyrus are crucial amodal semantic hubs. In the study of Simanova et al. (2014), participants had to judge the semantic category of target words in word reading and listening. Afterwards, as a language production task, there was a free recall session of the stimuli used in the categorization task. This study provided support for the involvement of the left inferior temporal cortex and frontal regions in the amodal representation of semantics. Hence, these three studies all supported the existence of amodal representations of conceptual properties of objects, although they didn’t completely converge on the specific neural localization, which may of course also be domain- and stimulus-dependent.

Interestingly, the studies discussed above have all tackled this debate from a monolingual perspective. However, nowadays more than half of the world population has knowledge of two or more languages, and can therefore be considered bilingual (Grosjean, 1992). Therefore, a second interesting question about the semantic system in the brain has arisen, which is about the extent to which neural representations of meaning overlap not only across modalities, but also across languages. The recruitment of a second, duplicate semantic network during L2 processing to represent almost the same knowledge as L1 would not be very parsimonious. And, indeed, theoretical models of bilingualism often assume shared semantics across languages, such as the revised hierarchical model (Kroll and Stewart, 1994), the BIA+ model (Dijkstra and van Heuven, 2002) and Green's convergence hypothesis (Green, 2003). However, this does not imply that the semantic representation of every concept should completely overlap across languages. Other models, like the distributed feature model (Van Hell and De Groot, 1998) or the model of Duyck and Brysbaert (2004) assume partially overlapping semantic representations between translation equivalents across languages, depending on specific characteristics of the concepts. They argued that the overlap in meaning, indexed by the number of shared semantic features, is larger for concrete translations, cognates and noun translations, relative to abstract translations, non-cognates and verb translations. In this view, the semantic representation of *apple* and *appel* for English-Dutch bilinguals would be shared to a larger degree than the representations of translation equivalents *justice* and *rechtvaardigheid*. Interestingly, there are also some empirical findings that suggest at least partly different semantic systems across languages. For instance, in Sahlin et al. (2005), English-Spanish bilinguals had to remember lists of semantically related words that were later probed for recognition. False recognition of semantic distractors was more frequent if study and test language were the same. This shows that semantic encoding may still be sensitive to the input language.

In addition, the idea of shared semantics that was implied in the early behavioral literature and theory on bilingualism (Kroll and Stewart, 1994) was also confirmed in the majority of classical neuroimaging studies. Hernandez et al. (2001), Klein et al. (1995) and Pu et al. (2001) for example reported overlap in semantic activation between L1 and L2 during word production. Likewise, Ding et al. (2003), lles et al. (1999) and Pillai et al. (2004) reported overlap in semantic activation between L1 and L2 during word comprehension. However, only a few studies have used MVPA to investigate neural overlap of semantic representations across languages, and those studies were always restricted to a single, specific modality (Buchweitz et al., 2012, Correia et al., 2014, Van de Putte et al., 2017, Yang et al., 2017). Buchweitz et al. (2012) were the first to apply MVPA to investigate semantic representations across languages. They used a word reading task that required translation equivalents in both languages to be read silently. Significant decoding accuracies were found across languages in the left inferior frontal gyrus, the left posterior superior temporal lobe, the postcentral gyrus, the occipital cortex and the left inferior parietal sulcus. To investigate auditory comprehension, Correia et al. (2014) used a word listening task that involved listening to the same words in both languages while judging the animacy of the words. They found significant decoding accuracies in the left anterior temporal lobe, the left angular gyrus, the left postcentral gyrus, the right posterior superior temporal gyrus, the right medial anterior temporal lobe, the right anterior insula and the bilateral occipital cortex. To investigate language production, in one of our own prior studies, we used a picture naming task that involved naming of the same concepts in both languages. We found significant decoding accuracies across languages in the bilateral middle occipital gyri, fusiform gyri and the inferior and middle temporal gyri (Van de Putte, et al., 2017). This suggests that semantic representations serving speech production in both languages overlap in the indicated brain areas. In these three studies, reliable prediction of the individual concepts was possible across languages. However, the identified brain regions differed across studies which each used different tasks and stimulus modalities (ie. Reading, listening and speech production). In addition, Yang et al. (2017) investigated semantic decoding of sentences across languages in addition to the decoding of individual semantic concepts across languages. The equivalent clustering of sentences in three languages provided evidence that neural representations of meaning are not only shared at the level of individual concepts, but also at higher-order levels.

Although these studies are very interesting for evaluating the extent to which semantic representations are shared across languages after semantic access from a specific language modality, they are not suited for determining the extent to which these language-independent semantic representations also converge across language modalities, because different tasks, experimental designs and participants were used. There is currently no comprehensive MVPA study that investigates the semantic neural representation across languages in bilinguals, incorporating different language tasks or modalities. Therefore, the goal of this study was to examine how the different languages are represented in the bilingual brain at a semantic level in different modalities, using a decoding approach. We assessed brain activation during L1 and L2 processing using tasks that tap selectively into the different language modalities, and investigated to what extent neural language overlap depends on the language modality at hand, within the same bilingual subjects. This approach does not only allow a cross-validation across different language modalities, contrasting language production with comprehension, it also assesses the integration or separation of L1 and L2 semantic representations. In the neuroimaging literature on bilingualism, such integrative research of language production and recognition systems across languages within the same participants does not yet exist.

## 1 Materials and methods

### 1.1 Participants

Twenty-two right-handed Dutch-French bilinguals (10 males, 12 females; mean age = 23.64, range = 20–27 years) participated in the study in exchange for a monetary compensation. The same participants who participated in the production part of the study reported in Van de Putte et al. (2017) also completed two other fMRI experiments. Of these 24 participants, 2 participants didn’t want to participate anymore and they were excluded from all analyses. All participants followed French courses at school from the age of 9 as part of the standard educational system in Flanders. Thirteen early simultaneous bilingual participants acquired Dutch and French from birth. They spoke French with their parents, Dutch at school and switched frequently between Dutch and French with their peers. Of the nine late sequential bilingual participants, three followed an additional high level French language education program, two had a job in which they often have to use both Dutch and French and four learned French at primary school but only have been using it occasionally since their graduation from secondary school. All recruited participants reported that they had normal vision and hearing abilities and were neurological and psychological healthy. All participants gave written informed consent prior to the experiments. The study was approved by the Ethical Committee of Ghent University hospital and all methods were carried out in accordance with the relevant guidelines and regulations.

### 1.2 Materials

Information about the participants’ self-assessed language proficiency, language switching frequency and the age of acquisition of both languages was measured with a language background questionnaire. To also obtain online measures of bilingual proficiency in Dutch and French, the LexTALE (Brysbaert, 2013, Lemhöfer and Broersma, 2012) and the Boston Naming test (BNT; Kaplan et al., 1983) were administered. The LexTALE is a comprehension-focused vocabulary test that gives a good indication of general Dutch and French proficiency. 70 existing words and 20 nonwords were used in the extended version of the Dutch LexTale (Lemhöfer and Broersma, 2012) and 56 existing words and 28 nonwords were used in the French Lextale (Brysbaert, 2013). The BNT is a 60-item picture-naming test that is assumed to measure word retrieval abilities and is more focused on production. The participants were asked to name the pictures in Dutch and French. The order of the languages in the LexTALE and the BNT was counterbalanced across participants (see Table 1 for results on these tests).

**Table 1:** Overview of language proficiency scores for the simultaneous and sequential bilinguals. The self-ratings are on a 5-point Likert scale and are averaged across listening, speaking, reading and writing.

**Group**	**Proficiency**	**Dutch (L1)**	**French (L2)**
**Simultaneous bilinguals*****(n=15)***	Lextale	59.85 (6.96)	43.21 (21.30)
	Boston Naming Test	51.53 (5.22)	43.67 (6.04)
	Self-Ratings	19.53 (1.30)	17.93 (1.75)
**High proficient sequential bilinguals*****(n=3)***	Lextale	64.99 (10.16)	61.31 (19.67)
	Boston Naming Test	56 (0)	41 (4.36)
	Self-Ratings	20 (0)	17.67 (2.52)
**Middle proficient sequential bilinguals*****(n=2)***	Lextale	69.15 (1.20)	43.75 (16.42)
	Boston Naming Test	53 (1.41)	33 (2.83)
	Self-Ratings	20 (0)	15 (1.41)
**Low proficient sequential bilinguals*****(n=4)***	Lextale	68.34 (3.04)	21.43 (3.57)
	Boston Naming Test	55 (2.94)	30.25 (7.85)
	Self-Ratings	20 (0)	13 (2.45)

### 1.3 Experimental procedure

To examine whether the semantic neural representations are shared across languages and modalities, the exact same 10 object concepts were used in three separate fMRI experiments that were administered on different days, each focusing on a specific task (picture naming, word reading and word listening). The sequence of the three tasks was counterbalanced across participants.

To examine whether the neural overlap between L1 and L2 semantic representations is common for the three language modalities, the 3 fMRI experiments were ran within the same participants. For picture naming, the dataset was the same as that used in our previous study (Van de Putte et al., 2017), so that comparisons of picture naming with word reading and word listening was possible within the same participants. All three fMRI studies were organized in 2 consecutive parts (a Dutch and a French part) and the order of languages was counterbalanced across participants.

The three different tasks were designed to be as dissimilar as possible in terms of sensory processing and task demands, but they all required access to the same underlying semantic representation of the concepts. In the picture naming task, participants were asked to produce the names of 10 concepts in Dutch and French (see Appendix for an overview of all pictures). All pictures were stored as 720 × 450-pixel images (18.1 × 11.3 visual degrees). Importantly, two maximally dissimilar images were selected per concept. Per participant, each image was associated with one language and this image-to-language assignment was counterbalanced across participants. This was done to assure that the activation when testing the individual concepts in one language could not rely on the visual characteristics of the depicted concept experienced when training in the other language.

The other two fMRI experiments focused on semantic representations accessed during language comprehension: in the word-reading task (requiring visual comprehension), participants had to read the same 10 concepts in silence and judge whether each concept was animate or inanimate (accessing semantics) by pushing the left or right button. In the word-listening task (requiring auditory comprehension), participants had to listen to the same 10 concepts while performing another categorization task in which they pushed the right or left button to answer the question: “Is the concept bigger or smaller in size than a football?”.

In order to ensure that the MVPA results reflect the underlying (shared) semantic representations and not merely the sensory similarities across languages and/or modalities, we selected two different images, two written translation equivalents without orthographic overlap and two spoken translation equivalents without phonological overlap, for each concept (e.g. horse; Dutch: paard, French: cheval) for each language. We minimized perceptual similarities in both the visual stimuli (view point and color between the two images of the same concept in the naming task and the letter size/font/color between the translation equivalents of the written words in the word-reading task) and the auditory stimuli (speaker gender and age between the translation equivalents of the spoken words in the word-listening task). The stimuli of a concept pair did not have any lexical overlap (overlapping phonemes and graphemes) across languages, as illustrated by the maximal levenshtein distance of 1.00 (SD=0) between Dutch and French translation equivalents (Levenshtein, 1965). Furthermore, the translation equivalents were also matched on word length (p>0.19) and familiarity (p>0.88).

The pictures and written words were presented for 1000 ms. Average pronunciation duration of the spoken words was 743 ms (range between 462 ms and 1033 ms). After stimulus presentation, a fixation cross was shown until the start of the next trial. The time between the response and the start of the next trial was jittered (mean = 2600 ms, range = 1000–5200 ms, in steps of 300 ms, distribution with pseudologarithmic density). In all three tasks, each language part included 5 experimental scan blocks of 60 trials. Within a block, each of the 10 concepts was randomly presented 6 times. The experimental blocks of each language part were preceded by a practice block (10 trials each) and in the naming task an additional familiarization block was included prior to the practice blocks to make sure that the participants named the pictures correctly.

### 1.4 fMRI data acquisition

Subjects were scanned with a 3 T Magnetom Trio MRI scanner system (Siemens Medical Systems, Erlangen, Germany). We used a standard 32-channel radio-frequency head coil. Participants were positioned head-first supine in the magnetic bore. To avoid motion artefacts, the participants were instructed not to move their heads. For each participant, the scanning procedure began with a high-resolution 3D structural scan, using a T1-weighted 3D MPRAGE sequence (TR = 2250 ms, TE = 4.18 ms, TI = 900 ms, acquisition matrix = 256 × 256 × 176, FOV = 256 mm, flip angle = 9 °, voxels resized to 1 × 1 × 1 mm). Next, whole brain functional images were collected using a T2*-weighted EPI sequence, sensitive to BOLD contrast (TR = 2000 ms, TE = 28 ms, image matrix = 64 × 64, FOV = 224 mm, flip angle = 80 °, slice thickness = 3 mm, distance factor = 17%, voxels resized to 3 × 3 × 3 mm, 34 axial slices). Per run, a fixed number of images (152) was acquired.

### 1.5 fMRI data preprocessing

Preprocessing and analysis of the fMRI data was performed using SPM8 software (Wellcome Department of Cognitive Neurology, London, UK). Reduction of T1 relaxation artefacts was pursued by exclusion of the first nine scans of all runs. The functional images were motion corrected with ArtRepair (Artifact Repair Toolbox v4), corrected for slice scan time differences and spatially realigned to their mean image by rigid body transformation. The anatomical image was normalized to the Montreal Neurological Institute (MNI) template brain image. The functional images were aligned with the high-resolution anatomical image to ensure an anatomically-based normalization. The low frequency artefacts in the time series data were removed using a high-pass filter with a cutoff at 128 s.

For each modality and separately for the two language parts, statistical analyses were performed on individual subjects’ data using the general linear model (GLM) in SPM8. Trials with incorrect semantic categorization were excluded from the analysis. The fMRI time series data were modelled by 10 different vectors, one for each semantic concept. All these vectors were convolved with a hemodynamic response function (HRF), as well as with the temporal derivative and entered into the regression model (the design matrix). Additionally, six motion parameters were added to the design matrix as regressors of no interest to account for variance related to head motion. The statistical parameter estimates were computed separately for all columns in the design matrix.

### 1.6 Whole brain MVPA analysis

To investigate the neural overlap between Dutch and French semantic representations, within and across the three tasks (naming, word reading and word listening), a multivariate decoding analysis was applied with the PyMVPA toolbox (Hanke et al., 2009). Multivariate decoding analyses were performed on the normalized but unsmoothed images to maximize the sensitivity to extract the full information in the spatial patterns, which might be reduced after smoothing (Misaki et al., 2013). Therefore smoothing was applied after multivariate decoding, prior to the second-level analyses with an 8 mm full-width half-maximum (FWHM) Gaussian kernel. A spherical whole brain searchlight with a radius of 3 voxels was applied to extract local spatial information from small brain spheres that carry information about the semantic concept (Kriegeskorte et al., 2006). The searchlight used the K Nearest Neighbours pattern classifier for this semantic classification (Hanke et al., 2009). Note that the use of other classifiers yielded similar results. More specifically, the classifier was trained to identify semantic activation of 10 concepts, associated with reading, listening to words or naming respective pictures, based on the neural pattern of brain activation elicited by reading/listening to /picture naming the same concepts in the other language. For instance, the classifier tried to predict neural activation triggered by the reading of the word *cheval* [horse] from the neural activation during reading (within-modalities) or listening/picture naming (across-modalities) of the translation equivalent *paard,* and vice versa.

Because one aim of the present paper was to investigate cross-lingual overlap, within tasks, we primarily focused on the across-language decoding analysis. For within-language analyses, the exact same stimuli (identical pictures, written words and spoken words) are by definition included, making it difficult to disentangle semantic activation from other overlapping visual, auditory or lexical features when applying MVPA. Across languages, visual and phonetical/acoustical similarities between the stimulus pairs of a concept and lexical similarities between the translation equivalents were maximally reduced in all three tasks to assure that classifier performance only reflected access to the shared semantic representation needed for the task in the two languages. The classifier was trained on the task-specific activation pattern associated with each of the 10 concepts in one language in four of the five blocks (training data set). Subsequently, this pattern classifier was used to classify the task-specific activation pattern for each of the 10 concepts in the corresponding fifth block of the other language (test data set). This procedure was repeated 5 times, so that each block could function as a test block once, while the other blocks were used as training blocks. Mean decoding accuracy maps across all five classifications were achieved for each participant in two directions (Dutch as training blocks and French as test block and vice versa). These across-language decoding accuracies were then averaged across the two directions, resulting in one mean decoding accuracy map across languages for each participant.

Additionally, in order to achieve our second aim, examining whether the semantic representations are shared across the three language modalities, MVPA was applied across modalities. Across modalities, we again only focused on the across-language decoding, because semantic overlap may by definition not be distinguished from lexical overlap in the within language decoding analysis, as this implies decoding activation after exposure to the same stimuli. For instance, a pattern classifier was trained on the activation pattern associated with the performance in L1 during the naming task, and then tested on how well it decoded the activation pattern associated with the performance in L2 during reading or listening. The underlying assumption was that the classifier would only be able to accurately predict which stimulus/concept was processed in the reading or listening task based on the activation in the naming task, if semantic representations overlap across these tasks. Across tasks there wasn’t any visual or auditory confound, because pictures, spoken words and written words of the same concepts relied on different sensory features.

#### 1.6.1 Within modalities second level analyses

To investigate how well decoding could be performed across all subjects, whole brain, voxel-by-voxel second-level statistical analyses were performed (Haynes et al., 2007). Whole brain searchlight analysis was interpreted as significant if decoding accuracies above chance level (10%) were observed. A one-sample t-test was used to reveal significant decoding of semantic concepts across languages, within the separate tasks. The significance thresholds of the group maps were all corrected for multiple comparisons at the cluster level (p < 0.05) and the voxel thresholds were either corrected for multiple comparisons (p < 0.05) or p < .001 uncorrected. Classification accuracies significantly above chance implied that the classifier was able to accurately predict which concept was named (or heard/read), whereas chance level performance implied that it was not possible to predict the concept that was named (heard/read). In all three tasks, brain regions that showed significant classifier prediction accuracy across languages indicate overlap between the semantic representations of L1 and L2.

#### 1.6.2 Across modalities second level analyses

Next, we investigated the language overlap of brain regions across pairs of tasks that each used different stimulus modalities. More specifically, we wanted to investigate whether it's possible to predict a concept in one modality/task based on the brain activity of that same concept in another modality/task and language. To reveal significant decoding of semantic concepts across each combination of tasks (naming-word reading, naming-word listening, word reading-word listening) a one-sample t-test was used to examine whether semantic representations overlap across the different language modalities. The one-sample t test and statistical thresholds were the same as for the within modalities second level analyses.

### 1.7 Region of interest analyses (ROI)

In addition to our whole brain approach, we also wanted to investigate whether regions that are reported to be involved in the previous literature on semantic processing in L1 word reading are also involved across L1 - L2 word reading, L1 - L2 production and L1 - L2 word listening. Hence, we additionally applied ROI analyses to distinguish whether neural representations within the same brain regions were different or the same for word reading, word listening and production. Our regions of interest were generated from an independent study of semantic processing of English words, relative to perceptual matching of meaningless symbols in monolingual English speakers. Paradigm details and results from this study have previously been reported by Seghier et al., 2010, Seghier et al., 2011. The 5 brain regions that were significantly involved in semantic association decisions on written words relative to perceptual association decisions on meaningless visual stimuli of equal complexity were: the left superior motor area, the left inferior frontal gyrus, the left middle temporal gyrus, the cerebellum and the left middle frontal gyrus (see Fig. 2). We used these regions of interest (ROI) associated with semantic processing of written words in a first language to test whether they were also activated in L2 word reading, production and word listening. Specifically, we tested whether activation could be predicted across L1 and L2 within word reading and/or word listening and production.

We tested the statistical significance of the group-level mean accuracy using a combination of permutation and bootstrap sampling methods (Stelzer et al., 2013). Specifically, we first permuted the stimulus labels of the 10 stimuli within each run and calculated the accuracies for each ROI for each participant using leave-one-run-out cross-validation. By repeating this procedure 100 times, we obtained 100 chance accuracies at the single participant level. Previous analyses have indicated that this number of repetitions is sufficient to achieve reliable estimation of false positive results (Stelzer et al., 2013). Next, we randomly sampled one of the chance accuracies from each participant and averaged these to obtain a chance group-level accuracy. This sampling (with replacement) was repeated 10000 times to create a group-level null distribution. For each ROI, the observed group-level accuracy was then compared to the group-level null distribution to obtain the associated *p*-value. A multiple comparison correction based on false discovery rate (P<0.05 FDR) was then applied at the group level on all P values associated with the 5 ROI's.

## 2 Results

### 2.1 Neural overlap across languages within tasks

**Fig. 1:**
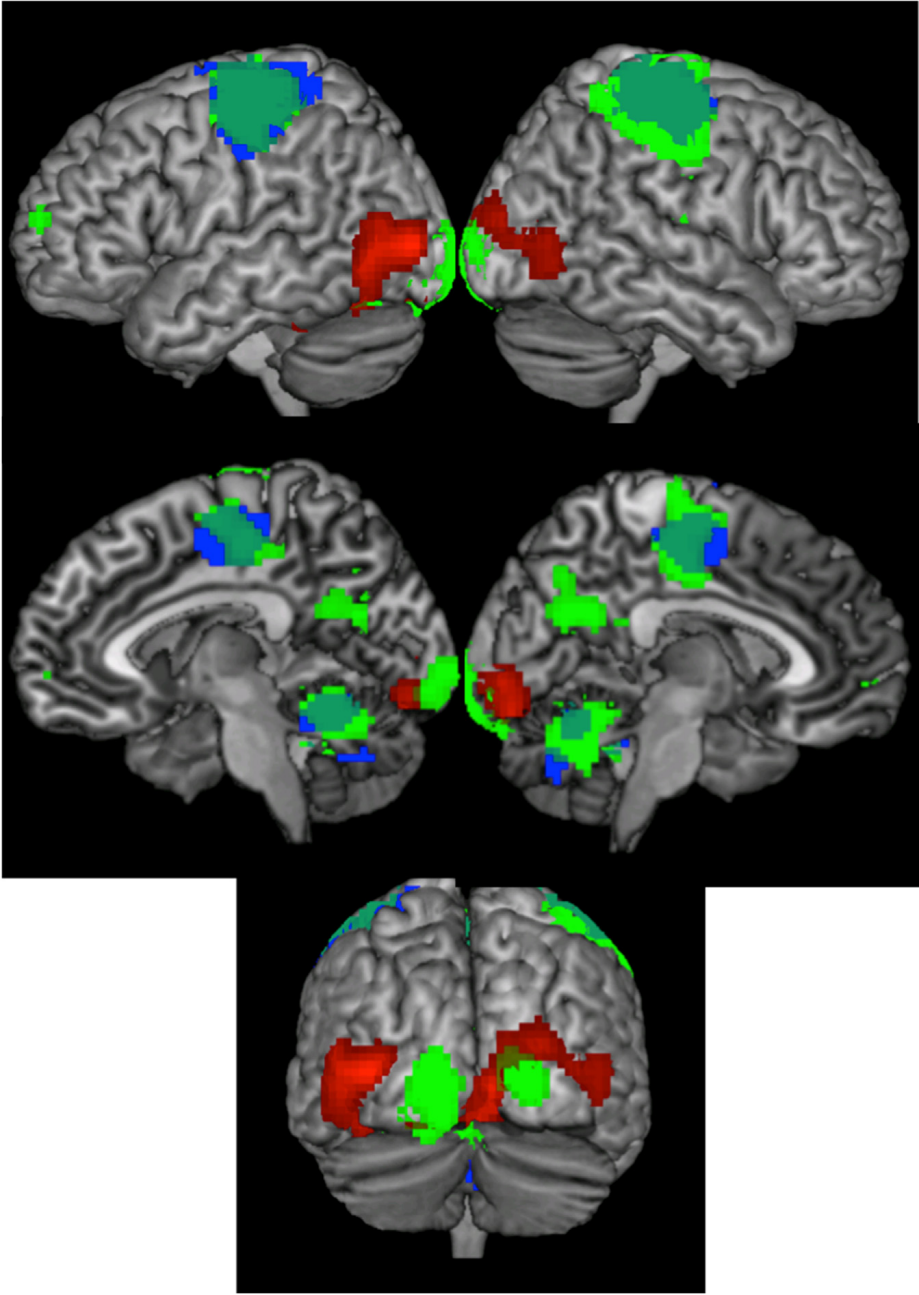
Results of the whole brain searchlight analysis showing discriminability between semantic concepts in the generalization across languages in naming (red), word reading (green) and word listening (blue). The color represents the t-values resulting from the group level analysis using a threshold of p < .001 at voxel level and a cluster level corrected for the whole brain at p < .05.

For picture naming, above chance decoding accuracies across languages were observed in the left middle occipital gyrus extending into the left fusiform gyrus, the right lingual gyrus extending into the right inferior temporal gyrus and left inferior temporal gyrus extending into the left hippocampus (Table 2; Fig. 1, red).

**Table 2:** Results of the across-language decoding analyses in production. All thresholds were FWE corrected in extent (Z scores in bold are also corrected in height).

Brain region	X	Y	Z	Z-score	Cluster size
Left middle occipital gyrus	−39	−85	4	**5.25**	635
Right lingual gyrus	9	−88	−2	**4.94**	773
Left inferior temporal gyrus	−42	−43	−26	3.85	113

For word reading, above chance decoding accuracies across languages were observed in the bilateral precentral gyrus extending into the postcentral gyrus, the left middle occipital gyrus, the left inferior occipital gyrus, the right calcarine sulcus, the bilateral cerebellum, the left inferior frontal gyrus, the left superior frontal gyrus, the right precuneus and the right rolandic operculum (Table 3; Fig. 1, green).

**Table 3:** Results of the across-language decoding analyses in word reading. All thresholds were FWE corrected in extent (Z scores in bold are also corrected in height).

Brain region	X	Y	Z	Z-score	Cluster size
Right precentral gyrus	42	−19	58	**6.32**	3998
Left middle occipital gyrus	−12	−97	4	**5.32**	380
Right Calcarine	24	−91	4	**5.22**	180
Vermis	6	−58	−29	4.64	893
Left inferior frontal gyrus	−39	20	19	3.82	118
Left superior frontal gyrus	−18	62	13	3.62	109
Right precuneus	0	−64	22	3.78	180

For word listening, above chance decoding across languages was observed in bilateral precentral gyri extending into the postcentral gyri, bilateral cerebella and the right rolandic operculum (Table 4; Fig. 1, blue).

**Table 4:** Results of the across-language decoding analyses in word listening. All thresholds were FWE corrected in extent (Z scores in bold are also corrected in height).

Brain region	X	Y	Z	z-score	Cluster size
Left precentral gyrus	−36	−19	58	**6.15**	1649
Left cerebellum	−15	−49	−20	**5.82**	597
Right postcentral gyrus	33	−28	55	**5.62**	1278
Right rolandic operculum	45	−13	22	4.13	101

### 2.2 Neural overlap across tasks and languages

We also applied MVPA across tasks to investigate whether shared neural representations across languages are involved across modalities. This would provide strong evidence for an integrative semantic neural representation across modalities, because stimuli were not sensory or phonologically confounded across tasks. Across modalities and languages, only significant encoding of semantic information was observed for the decoding analyses across production and word reading, namely in the left lingual gyrus (Table 5). Lowering the voxel-level threshold to p < .005 (instead of *p* < .001) did not result in any additional regions across the other tasks.

**Table 5:** Results of the across-language decoding analyses across modalities. The threshold was FWE corrected in extent and height.

Modality	Brain region	X	Y	Z	z-score	Cluster size
Production – word reading	Left lingual gyrus	−18	−82	−2	**4.90**	240

### 2.3 Region of interest analyses (ROI)

**Fig. 2:**
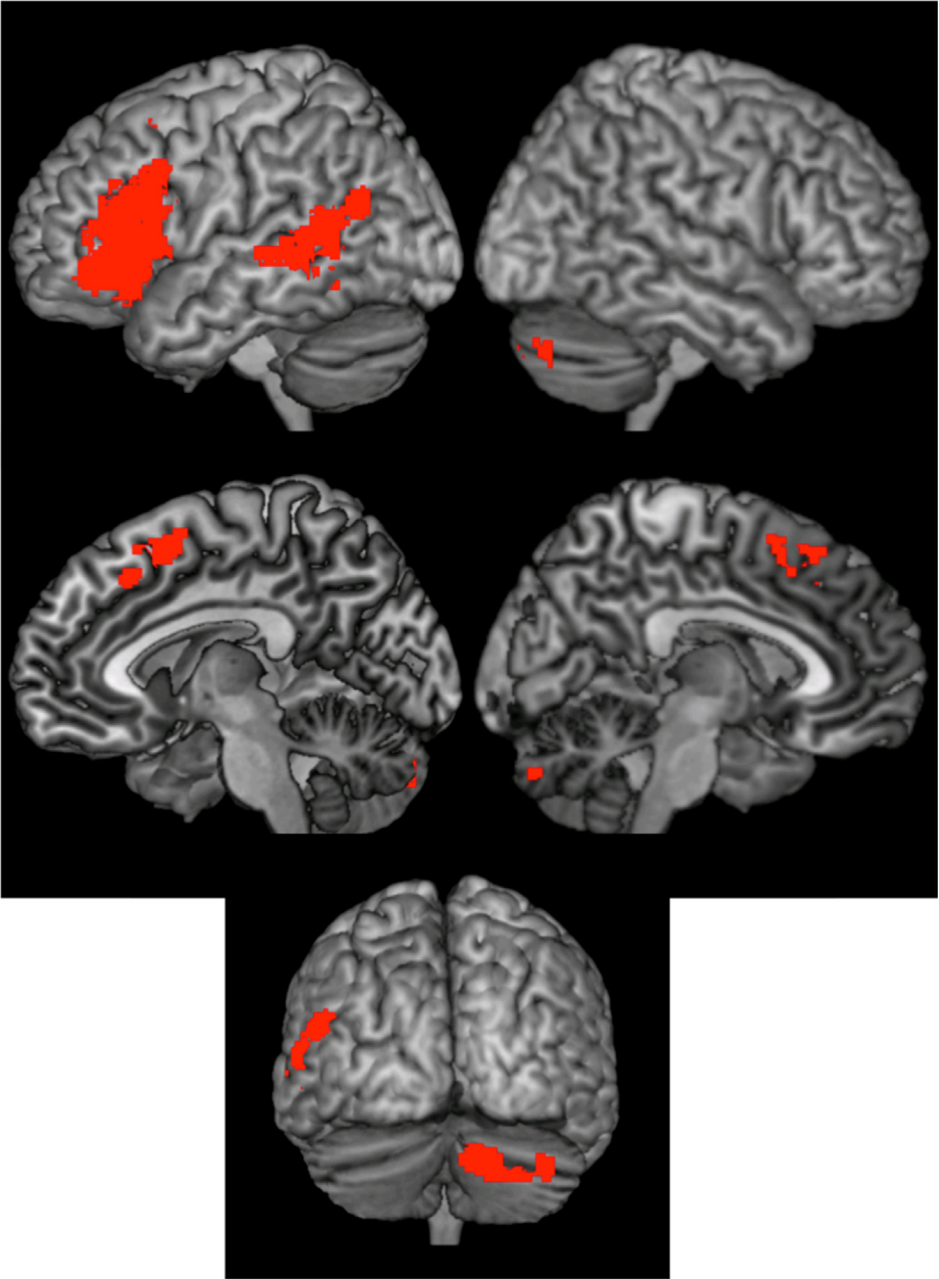
Regions of interest (ROI's) associated with semantic processing of written words in a first language (Seghier et al., 2010, Seghier et al., 2011, Seghier and Price, 2012, Seghier and Price, 2013).

Within five regions of interest (Fig. 2) that have previously been associated with the representation of semantics in L1 word reading, we investigated whether there was also evidence of the same semantic representations across L1L2 word reading, across L1L2 production and across L1L2 word listening (Table 6). Three of the five ROI's (the left superior motor area, the left inferior frontal gyrus and the left middle temporal gyrus) showed significant across-language decoding accuracies in our word reading task after FDR correction. None of these ROI's however showed above-chance prediction accuracies in the decoding across languages in the word listening task or the production task. Hence, these regions seem to be recruited specifically for the language independent semantic representation during word reading, and could not be generalized to the language independent semantic representation during word listening or production.

**Table 6:** Across languages Region of interest (ROI) analyses within the three modalities.

Brain region	Coordinates	Task	P
Left inferior frontal gyrus	−45 23 12	Word reading across languages	0.0002**
		Word listening across languages	0.3787
		Production across languages	0.4116
Left middle temporal gyrus	−56 −44 4	Word reading across languages	0.0048*
		Word listening across languages	0.3008
		Production across languages	0.1564
Cerebellum	20 −78 −35	Word reading across languages	0.3338
		Word listening across languages	0.8215
		Production across languages	0.6179
Left superior motor area	−3 16 53	Word reading across languages	0.0097*
		Word listening across languages	0.1211
		Production across languages	0.2327
Left middle frontal gyrus	−27 13 52	Word reading across languages	0.1411
		Word listening across languages	0.7881
		Production across languages	0.0664

***p<0.001.

* p<0.05;

** p<0.01;

## 3 Discussion

In the present study, we used MVPA to investigate the neural overlap between semantic representations tapped into by both languages of Dutch-French bilinguals, and the overlap of these representations across language modalities. MVPA was used because of the advantage of this technique to deduct cognitive representations from brain signals (Haxby et al., 2001, Haynes et al., 2007). This is the first study to examine whether decoding of individual semantic concepts across languages was possible across tasks (that used different stimulus modalities), within the same (bilingual) individuals.

In this group of mainly high proficient bilinguals, the results showed that encoding of semantic information was possible across languages, for each of the three tasks. It was possible to identify the picture/word named, read or heard in one language based on the brain activity observed while naming, reading or listening the picture or word in the other language. However, the brain regions that predicted commonality in across-language representations differed across tasks. For picture naming, the across-language overlap was identified in regions associated with object recognition: the bilateral middle occipital and fusiform regions extending into the inferior temporal regions. A first interesting type of regions was observed in the across-language overlap for word reading and word listening. More specifically, significant decoding across languages in word reading was possible in visual processing regions (left middle occipital gyrus extending into the left inferior occipital gyrus, the right calcarine), and in regions associated with higher cognitive functions (the left inferior frontal gyrus, the left superior frontal gyrus and the right precuneus). For word listening, the across-language overlap was identified in the rolandic operculum, which was something surprising given that this region's role for language processing was mostly linked to phonological, rather than semantic processing (Tonkonogy and Goodglass, 1981, Vigneau et al., 2006). Together, the results from these across-language analyses show that all modalities tap into neural representations of semantics that at least partly overlap across languages. Therefore, they are consistent with theoretical models of bilingualism that posit such shared semantics across languages, such as the revised hierarchical model (Kroll and Stewart, 1994), the BIA+ model (Dijkstra and van Heuven, 2002), Green's convergence hypothesis (Green, 2003) and the distributed feature model (Van Hell and De Groot, 1998; for a similar model, see Duyck and Brysbaert, 2004).

In addition, for word listening, and also for word reading, the second type of regions that showed across-language overlap was of less theoretical significance because it concerned regions associated with sensorimotor processing: the bilateral precentral gyrus extending into the postcentral gyrus and the bilateral cerebellum. The involvement of these sensorimotor regions should be interpreted with care in word reading and word listening, because the semantic category required the same button response for each language. In word reading the left button was for example always associated with the judgment *animate* and the right button with *non-animate* or vice versa. Similarly, in word listening the left button was always associated with the judgment *bigger than a football* and the right button was always associated with *smaller than a football,* or vice versa. Hence for the sensorimotor regions it was not possible to distinguish whether significant decoding accuracies could be attributed to overlapping semantic representations or sensorimotor representations.

The involvement of inferior frontal and occipital regions in our word reading task are in line with the results of Buchweitz et al. (2012) who also applied decoding to investigate semantic neural overlap across languages in word reading. The contribution of the inferior frontal gyrus and the left superior frontal gryrus in the word reading task was furthermore consistent with the review of Binder et al. (2009). They showed that the inferior and superior frontal gyri are typically involved during semantic processing in a broad range of comprehension studies. The engagement of occipital regions and the calcarine in our word reading and production task fits within the embodiment idea, because occipital regions are not only shown to be activated during visual stimulation, but also during tasks that didn’t use visual stimuli. Therefore mental imagery as part of the semantic representations could be a possible explanation (Klein et al., 2000, Lambert et al., 2002). The concept cat for example may include visual features (four legs, tail, whiskers), acoustic features (meows) and emotional aspects (love or disgust) that are dependent on the individual experience with the concept. We only used concrete concepts that are all imaginable, which in accordance with the embodiment view may imply conceptual representations that might differ dependent on the individual experiences that are associated with the concepts throughout life experiences (Kiefer and Pulvermüller, 2012). Therefore the comparison with conceptual representations of abstract words across languages and modalities within the same subjects would be of added value in this research field. As shown by Wang et al. (2010) concrete concepts could for example be associated more profoundly with perceptual regions than abstract concepts, because concrete concepts are more imaginable than abstract concepts.

Additionally, we applied ROI analyses on five brain regions that have previously been associated with the representation of semantics in L1 word reading to investigate whether these regions also generalize to L2 word reading and production and word listening. In our word reading task, we replicated the involvement of the left superior motor area, the left inferior frontal gyrus and the left middle temporal gyrus in the decoding across languages. We could therefore assume that these regions that are reported to be involved during semantic processing in L1 word reading generalize to L2 word reading. However, none of these ROI's was significant in the decoding across languages within word listening, nor in the decoding across languages within production. Hence, the activated brain regions for semantics vary depending on the language modality involved and the specific task characteristics that are associated with language modality. This might explain the varying brain regions identified in different studies, because depending on the experimental task, different aspects of semantics could result in the involvement of different brain regions. These results provide evidence for distributed semantic models in which concepts are flexible, distributed in the brain, and dependent on the specific modality at hand (Barsalou et al., 2003, Kiefer and Pulvermüller, 2012, Tyler and Moss, 2001, Musz and Thompson-Schill, 2015).

In addition to the question whether semantic representations overlap across languages, the other aim of the present paper was to investigate whether semantic representations also overlap across both languages ànd modalities. Importantly, in this analysis lexical, sensory and motor overlap is ruled out, as there wasn’t any lexical confound across languages (overlapping graphemes and phonemes were minimal between the translation equivalents of the same concepts) and there wasn’t any sensory or motor confound across modalities (different tasks were used across modalities that relied on different sensory features and required different motor responses). This analysis showed that across-language decoding was only possible across production and word reading in the left lingual gyrus. Hence, across modalities, it was only possible to identify the picture the participant was naming in one language based on the neural activation patterns in the left lingual gyrus observed during the presentation of the equivalent written word in the other language and vice versa. This suggests that the lingual gyrus might play a crucial role in the integration of language independent semantic information across modalities (at least across production and word reading). The role of the lingual gyrus in semantic integration across modalities converges with the findings of Musz and Thompson-Schill (2015), who argued that the lingual gyrus is an important semantic hub across different semantic contexts. More specifically, they showed that variation of neural patterns in the lingual gyrus reflects variation in the conceptual processing of concepts across variations in their semantic contexts. Despite the common brain regions that are involved in the across-modality decoding analyses across word reading and production, no significant brain regions were observed in the decoding across word reading and word listening and the decoding across production and word listening. These findings support the idea of both a-modal and modality-dependent semantic representations that nevertheless overlap across languages (Bonner et al., 2013). Note that we also ran decoding analyses across modalities, but within-languages. This was not our primary focus as such analyses by definition imply a confound of lexical overlap: within-languages, the concepts do not only share semantics, but also lexical information (orthography, phonology). Hence, neural activation identified by significant decoding may then possibly represent lexical, rather than semantic activation. But even then, only decoding between production and word reading in L2 was significant in the left rolandic operculum. Across word reading - word listening and across production - word listening decoding was neither significant within L1, nor within L2. Hence, this also supports the notion that even within-languages semantics activation is largely modality-specific.

Although the shared neural activation in decoding across languages ànd modalities was limited, the evidence for an amodal semantic hub like the lingual gyrus in our analyses is in line with the results of Fairhall and Caramazza (2013) and Simanova et al. (2014), who also adopted a similar decoding approach and also provided evidence for the existence of amodal semantic representations. They didn’t, however, completely converge on the specific neural localization, which may of course also be domain- and stimulus-dependent. Simanova et al. (2014) argued that these amodal representations are located in the left inferior temporal cortex and frontal regions, while Fairhall and Caramazza (2013) argued for the localization in the precuneus and the posterior middle/ inferior temporal gyrus. An important difference was however that the current study tried to predict individual semantic concepts across modalities, whereas the studies of Fairhall and Caramazza (2013) and Simanova et al. (2014) assessed the representation of broad semantic categories across modalities.

In the literature about semantic organization, an interesting debate has also arisen about whether or not semantic representations are more local than distributed. According to the local view, a concept is represented as a single node within a unitary semantic network (Bowers, 2009, Collins and Quillian, 1969, Kiefer and Pulvermüller, 2012). In these localist models, meaning is represented by fixed unitary concept nodes that are connected within a semantic network. To compensate for the absence of conceptual flexibility in these localist models, distributed semantic models have suggested that concepts are represented by multiple representational units that can be adjusted through experiences. These models assume that meaning results from the interactions of neurons through synaptic connections, in which the meaning of a concept (“dog”) arises due to the activation of a combination of semantic features (barks, animal, tail) or processing units (Barsalou et al., 2003, Kiefer and Pulvermüller, 2012, McClelland and Rogers, 2003, Smith et al., 1974, Tyler and Moss, 2001). Although this is an interesting question that also tackles the way semantics are represented, we can’t really distinguish the two possibilities in the current study because we didn’t investigate whether the individual concepts are represented by separate neurons that reflect local representations for each concept or separate neural networks that represent multiple representation units for each concept.

Future research may also clarify to what extent the current findings interact with individual variables like age of language acquisition, and proficiency. For instance, recruiting a more homogeneous subject group of highly proficient early bilinguals could have resulted in the involvement of additional significant brain regions that showed neural overlap (Indefrey, 2006, Stowe and Sabourin, 2005) across languages. Or even modalities, because practice within a given language may also affect cross-modal integration of representations. Of course, assessing such individual difference variables requires recruitment of much larger participant groups, and would therefore also interfere with the full-factorial within-subject design across languages and modalities that was adopted here.

To conclude, our results provide evidence for at least partially language-independent semantic representations that rely on a distributed semantic network that includes both an a-modal, integrative representation and modality specific representations.
